# Artificial Olfactory Neuron for an In‐Sensor Neuromorphic Nose

**DOI:** 10.1002/advs.202106017

**Published:** 2022-04-15

**Authors:** Joon‐Kyu Han, Mingu Kang, Jaeseok Jeong, Incheol Cho, Ji‐Man Yu, Kuk‐Jin Yoon, Inkyu Park, Yang‐Kyu Choi

**Affiliations:** ^1^ School of Electrical Engineering Korea Advanced Institute of Science and Technology (KAIST) 291 Daehak‐ro, Yuseong‐gu Daejeon 34141 Republic of Korea; ^2^ Department of Mechanical Engineering Korea Advanced Institute of Science and Technology (KAIST) 291 Daehak‐ro, Yuseong‐gu Daejeon 34141 Republic of Korea

**Keywords:** electronic nose (E‐nose), electronic sommelier, neuromorphic system, olfactory neuron, spiking neural network (SNN)

## Abstract

A neuromorphic module of an electronic nose (E‐nose) is demonstrated by hybridizing a chemoresistive gas sensor made of a semiconductor metal oxide (SMO) and a single transistor neuron (1T‐neuron) made of a metal‐oxide‐semiconductor field‐effect transistor (MOSFET). By mimicking a biological olfactory neuron, it simultaneously detects a gas and encoded spike signals for in‐sensor neuromorphic functioning. It identifies an odor source by analyzing the complicated mixed signals using a spiking neural network (SNN). The proposed E‐nose does not require conversion circuits, which are essential for processing the sensory signals between the sensor array and processors in the conventional bulky E‐nose. In addition, they do not have to include a central processing unit (CPU) and memory, which are required for von Neumann computing. The spike transmission of the biological olfactory system, which is known to be the main factor for reducing power consumption, is realized with the SNN for power savings compared to the conventional E‐nose with a deep neural network (DNN). Therefore, the proposed neuromorphic E‐nose is promising for application to Internet of Things (IoT), which demands a highly scalable and energy‐efficient system. As a practical example, it is employed as an electronic sommelier by classifying different types of wines.

## Graphical abstract

A neuromorphic module of an electronic nose (E‐nose) is demonstrated by hybridizing a semiconductor metal oxide (SMO) and a single transistor neuron (1T‐neuron). By mimicking a biological olfactory neuron, it can perform gas detection and spike encoding simultaneously for in‐sensor neuromorphic functioning. It is helpful to realize a highly scalable and energy‐efficient E‐nose for mobile gas sensors and IoT applications.

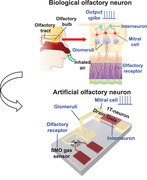

## 1 Introduction

**Figure 1:**
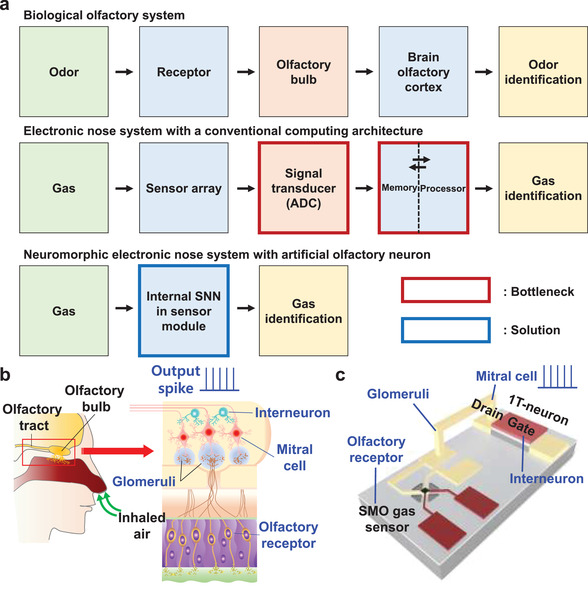
Biological olfactory system and proposed neuromorphic E‐nose system with artificial olfactory neuron. a) Block diagram showing a biological olfactory system, a conventional DNN‐based E‐nose with a conventional processor, and the proposed spiking neural network (SNN) based neuromorphic E‐nose with an artificial olfactory neuron. Limits of hardware cost and energy consumption can be resolved by using an internal SNN in a sensor module. b) A biological olfactory system composed of various olfactory neurons including olfactory receptors, Glomeruli cells, mitral cells, and interneurons in the olfactory bulb. c) A proposed artificial olfactory neuron module composed of a SMO gas sensor and a MOSFET‐based 1T‐neuron. The SMO gas sensor acts as an olfactory receptor that detects odorants. The metal interconnection serves as a Glomeruli that transports signals from the olfactory receptors to the mitral cell. The drain of the 1T‐neuron acts as a mitral cell that receives signals from the olfactory receptors and transmits the spike signals to the olfactory cortex. The gate of the 1T‐neuron serves as an interneuron that inhibits neuronal firing laterally.

Developing a portable and personalized electronic olfactory system has become increasingly important in the era of the Internet of Things (IoT). Specifically, a palmtop‐sized mobile gas sensor can be used to monitor the air quality levels of contaminants and fine dust indoors and outdoors as well as food quality, and for healthcare applications such as breath‐based early diagnosis of diseases.^[^ ^1^, ^2^, ^3^, ^4^, ^5^, ^6^ ^]^ In addition, mobile gas sensors are widely used in industrial fields to enhance the production yields and to ensure workers’ safety from toxic gases.^[^ ^7^, ^8^, ^9^ ^]^ Recently, an electronic nose (E‐nose) that uses a sensor array and artificial intelligence system to mimic a biological olfactory system has attracted strong interest in the research community.^[^ ^10^, ^11^, ^12^, ^13^, ^14^ ^]^ A biological olfactory sensing system detects various smells by processing signals from a cluster of olfactory neurons, as shown in **Figure** **1**a,b. When olfactory receptors sense an odorant, the chemical reactions between them trigger electrical signals as an output. The signals are then transmitted through the glomeruli to the olfactory bulb, where mitral cells and interneurons are located. Signal preprocessing is performed in the olfactory bulb, and then the preprocessed signals are transmitted to the brain olfactory cortex to identify the odor.

Inspired by the biological olfactory sensing system, an E‐nose usually consists of a sensor array, a transducer composed of electronic circuits for signal pre‐processing, such as an analog‐to‐digital converter (ADC), and a processor based on von Neumann computing that operates with the aid of data analysis software.^[^ ^15^, ^16^, ^17^ ^]^ However, when data are transferred from the sensor to the processors, hardware cost and energy consumption can be increased by the number of required components, such as the conversion circuits used for the signal preprocessing.^[^ ^18^, ^19^, ^20^ ^]^ In addition, implementing multistage data processing imposes a heavy workload on hardware systems with a von Neumann architecture, which is composed of a few subcomponents, e.g., a central processing unit (CPU), memory, etc. The input and output data bus between memory and processors also inevitably add hardware cost and power consumption. Ultimately, the limiting factor to realizing a portable gas monitoring device for IoT applications is whether all the components can be incorporated into a hardware system that has small size and low power.

It should be noted that biological sensory systems, including the olfactory system, process massive amounts of data with the aid of spike‐based signal transmission, which can minimize power consumption. To imitate this biological sensory system and to improve the energy efficiency compared to a conventional von Neumann architecture, a hardware‐based spiking neural network (SNN) with a neuromorphic architecture is considered a promising solution.^[^ ^21^, ^22^, ^23^, ^24^ ^]^ To apply the SNN to a sensory system, signals collected from the environment need to be transformed into spike forms; however, this cannot be realized using conventional sensors. A circuit, which converts a sensory signal collected from a conventional sensor into a neuronal spike signal, can be used. However, it is composed of oversized and complex components including an op‐amp or a Schmitt trigger, which are accompanied with high power consumption and expensive hardware cost. These are the limiting factor to realize a portable gas monitoring device for IoT applications. Recently, artificial sensory neurons that can simultaneously perform sensing and spike encoding for in‐sensor neuromorphic functioning have been proposed in the fields of visual sensing and tactile sensing systems (Table S1, Supporting Information).^[^ ^25^, ^26^, ^27^ ^]^ By using the internal neural processing in a sensor module, they have been able to convert continuous analog signals into a discrete spike pattern without conversion circuits including an ADC, and other hardware components used for von Neumann computing. As a result, they have reduced hardware cost and power consumption. While systems have been developed to perform visual or tactile sensing, an E‐nose system that harnesses an internal neural processing unit has not yet been proposed.

In this study, we propose an artificial olfactory neuron module that can simultaneously perform gas sensing and spike encoding. This artificial olfactory neuron module is composed of a chemoresistive gas sensor made of a semiconductor metal oxide (SMO) and a single transistor neuron (1T‐neuron) made of a metal‐oxide‐semiconductor field‐effect transistor (MOSFET). This artificial olfactory neuron module acts as an input neuron for the SNN by imitating the characteristics of biological olfactory neurons. It modulates the spiking frequency depending on a targeted gas without a transducer or a conversion circuit. In addition, extra computing units including the CPU and memories, which are indispensable for the von Neumann architecture, are not necessary either owing to the neuromorphic architecture. Both ON‐ and OFF‐type sensory neuron responses are realized by the artificial olfactory neuron module in different manners.^[^ ^28^ ^]^ The SMO‐based gas sensor can be miniaturized, with low‐cost and high sensitivity appropriate for mobile and personalized gas sensing.^[^ ^29^, ^30^, ^31^ ^]^ The 1T‐neuron has important benefits in terms of hardware cost and power consumption compared to a conventional circuit‐based CMOS neuron.^[^ ^32^, ^33^, ^34^ ^]^ In addition, the gate electrode of the 1T‐neuron can control the lateral inhibition of the artificial olfactory neuron, which can identify odorants with improved accuracy and energy efficiency by virtue of adaptation and distinctive signal contrast. By combining SMO gas sensors and 1T‐neurons, a highly scalable and energy‐efficient E‐nose module is proposed for a mobile sensor system and IoT application. Beyond device‐level characterization, we performed experiment‐based software simulations to classify four gases (NH_3_, CO, acetone, NO_2_). Furthermore, it is also utilized to distinguish different types of red wines (“Shiraz” and “Merlot”), demonstrating that the proposed artificial olfactory neuron module can act as an electronic sommelier.

## 2 Results and Discussion

### 2.1 Structure of the Artificial Olfactory Neuron Module

A SMO gas sensor and a 1T‐neuron were connected serially to implement the artificial olfactory neuron module, as shown in Figure 1c. The SMO gas sensor, analogous to a variable resistor, fulfilled the role of an olfactory receptor in a biological olfaction system, which is responsible for detecting odorants.^[^ ^35^, ^36^ ^]^ The metal interconnection between the SMO gas sensor and 1T‐neuron serves as the glomeruli, which transports signals from the olfactory receptors to the mitral cell. The drain of the 1T‐neuron behaves like the mitral cell, which receives signals from the olfactory receptors and transmits the spike signals to the brain olfactory cortex.^[^ ^37^, ^38^ ^]^ The gate of the 1T‐neuron controls the inhibition of the olfactory neuron. In the biological olfactory system, lateral inhibition of the mitral cell is performed by an interneuron in the olfactory bulb.^[^ ^39^, ^40^, ^41^ ^]^ Lateral inhibition allows adaptation, which is important for desensitizing the response to noxious odorants and holding one's breath. Note that humans undergo this adaptation daily when the nose becomes blind to strong smells. It also enhances sensory perception by creating a signal contrast and enhances energy efficiency by inhibiting the useless neurons. It is noteworthy that a parasitic capacitor is connected in parallel with the 1T‐neuron and hence the spiking frequency of the artificial olfactory neuron module can be modulated similar to a biological mitral cell.^[^ ^42^, ^43^ ^]^

### 2.2 Characteristics of the SMO Gas Sensor

The SMO gas sensor uses a semiconducting metal oxide as a sensing material. The working principle of the SMO gas sensor is based on the changes in the electrical resistance of the sensing material.^[^ ^44^ ^]^ In the presence of oxygen in the ambient, an electron‐depleted region is generated on the surface of the metal oxide because free electrons are extracted when the oxygen is ionosorbed on the surface.^[^ ^45^, ^46^ ^]^ When a gas is adsorbed onto the surface of the sensing material, the electron‐depleted region is changed by the reaction between the gas and the ionosorbed oxygen. This change in the electron‐depleted region results in a resistance change of the metal oxide. Using this response, the SMO gas sensor can detect various gases. For example, when a reducing gas (e.g., CO) reacts with an n‐type metal oxide (e.g., SnO_2_), the resistance of the metal oxide decreases. Conversely, an oxidizing gas (e.g., NO_2_) increases the resistance of metal oxides.^[^ ^47^ ^]^

**Figure 2:**
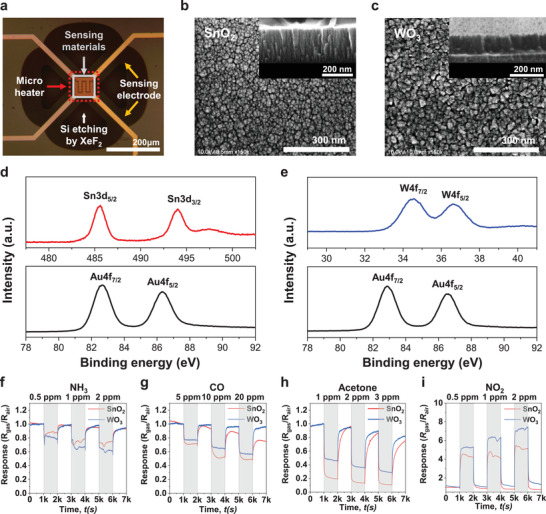
Characteristics of the fabricated SMO gas sensor. a) Microscopic image of the SMO gas sensors using bridge‐type microheater platform. Au‐coated metal oxide thin films (SnO_2_ and WO_3_) on the microheater platform were used for the sensing materials of the SMO gas sensors. b, c) SEM images with top and cross‐sectional (inset) views of the nanocolumnar SnO_2_ and WO_3_ thin films deposited by the glancing angle deposition method. d,e) Sn3d, W4f, and Au4f XPS spectrum of Au‐coated SnO_2_ and WO_3_ oxide films for the SMO gas sensors. f–i) Dynamic responses of the SnO_2_ and WO_3_ gas sensors to 4 gas species: NH_3_, CO, acetone, and NO_2_ gas. Each gas sensor harnesses a bridge‐type microheater. For heat‐up, electrical power of 12.9 mW was applied to the microheater. The response (*R*_gas_/*R*_air_) decreased for NH_3_, CO, and acetone, whereas it increased for NO_2_. Shaded and unshaded areas represent time intervals exposed to gas and to air, respectively.

Since adsorption reactions of a gas on a metal oxide surface are typically activated at high‐temperature conditions (200 °C to 400 °C), SMO gas sensors have been widely used as a microheater platform to efficiently transfer heat to the sensing material.^[^ ^48^, ^49^ ^]^ In this study, as shown in **Figure** **2**a, a low‐power, bridge‐type microheater platform was used to transfer the heat to the SMO gas sensors.^[^ ^50^ ^]^ SnO_2_ and WO_3_ were used as the sensing materials for the SMO gas sensors and were deposited on the microheater platform in the form of nanocolumnar films using glancing angle deposition via RF sputtering. Also, for a catalytic effect, Au nanoparticles were additionally coated on the metal oxide thin films. The detailed fabrication process of the SMO gas sensors is shown in Figure S1 (Supporting Information). Scanning electron microscopy (SEM) images of the nanocolumnar SnO_2_ and WO_3_ films are shown in Figure 2b,c, respectively. The surface property of the sensing materials was investigated by X‐ray photoelectron spectroscopy (XPS) analysis. As shown in Figure 2d,e, the peaks of Sn3d and Au4f for the Au‐coated SnO_2_ gas sensor and the peaks of W4f and Au4f for the Au‐coated WO_3_ gas sensor were observed clearly.

In this study, four target gases (NH_3_, CO, acetone, and NO_2_) were used to measure the characteristics of the fabricated SMO gas sensors, and microheater power (*P*) of 12.9 mW was applied during the gas tests. The responses of the gas sensors were quantified as the ratio of resistance in the gas environment to resistance in the air environment (*R*_gas_ */R*_air_). The dynamic responses of the gas sensors to the target gases are shown in Figure 2f‐i. Since SnO_2_ and WO_3_ are n‐type metal oxides, their resistances decreased when they reacted with reducing gases (NH_3_, CO, acetone). On the other hand, when they reacted with an oxidizing gas (NO_2_), their resistances increased.

### 2.3 Characteristics of a 1T‐Neuron

**Figure 3:**
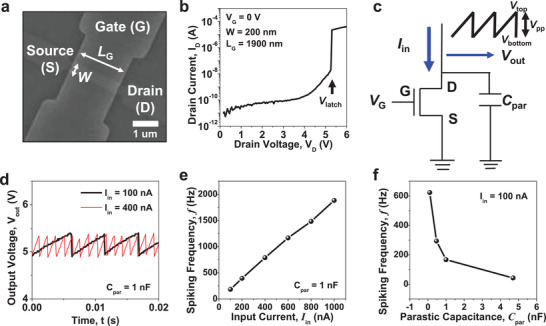
Characteristics of the fabricated 1T‐neuron. a) Scanning electron microscopy (SEM) image of the fabricated 1T‐neuron. b) Output characteristics (*I*_D_–*V*_D_) of the fabricated 1T‐neuron. Large current was abruptly flown when the drain voltage (*V*_D_) reached the latch up voltage (*V*_latch_) by a phenomenon of a single transistor latch (STL). This allows firing of the integrated charges during neuron operation. c) Measurement scheme for neuron operation. Constant input current (*I*_in_) was applied to the drain electrode and output voltage (*V*_out_) was measured at the same drain electrode. d) Spiking characteristics (*V*_out_–*t*) of the fabricated 1T‐neuron. The typical spiking characteristics of a leaky integrate‐and‐fire (LIF) neuron were achieved. e) Measured spiking frequency (*f*) as a function of the input current (*I*_in_) in the fabricated 1T‐neuron. *f* was increased as *I*_in_ was increased because the charging speed to the parasitic capacitor was boosted. f) Measured *f* as a function of *C*_par_. *f* was reduced as *C*_par_ was increased because the incremental speed of *V*_out_ was retarded.

A 1T‐neuron can mimic the neuronal leaky integrate‐and‐fire (LIF) function by using the single transistor latch (STL) phenomenon.^[^ ^32^, ^33^, ^34^ ^]^ In this study, a 1T‐neuron was fabricated with a MOSFET structure on a silicon on insulator (SOI) substrate. For the starting wafer, a p‐type (100) SOI wafer was used. First, active silicon areas were patterned by photolithography and subsequent plasma etching. Afterward, ion implantation with boron followed by rapid thermal annealing (RTA) was performed for body doping. Sequentially, a gate dielectric and n^+^ poly‐crystalline silicon (poly‐Si) were deposited and patterned for a gate electrode (G). Next, ion implantation with arsenic and another RTA was performed to form the source/drain (S/D). Finally, interlayer dielectric (ILD) deposition and metallization with Ti/TiN/Al were performed. The fabricated 1T‐neuron had a channel width (*W*) of 200 nm and a gate length (*L*_G_) of 1900 nm, as shown in the SEM image in **Figure** **3**a. The fabrication details are presented in Figure S2 (Supporting Information).

Figure 3b shows the output characteristics of the fabricated 1T‐neuron, represented by the drain current versus drain voltage (*I*_D_–*V*_D_). When a gate voltage (*V*_G_) of 0 V was applied, a large amount of *I*_D_ abruptly flowed beyond a certain *V*_D_, which is called the latch‐up voltage (*V*_latch_). This is known as the STL phenomenon, and allows for instant firing of the neuron.^[^ ^33^, ^34^ ^]^ It should be noted that when a *V*_G_ of 2 V was applied, *I*_D_ flowed regardless of the *V*_D_ because the *V*_G_ was higher than the threshold voltage (*V*_T_) of the MOSFET, and the MOSFET was turned ON by channel formation, as shown in Figure S3a (Supporting Information). This binary current level, which is controlled by the gate voltage, will be used for the inhibitory function of the 1T‐neuron and artificial olfactory neuron module.

To operate the 1T‐neuron, a constant input current (*I*_in_) was applied to the drain electrode, and output voltage (*V*_out_) was measured at the same drain electrode, as illustrated in Figure 3c. Note that a parasitic capacitor with a certain amount of capacitance (*C*_par_) was connected parallelly to the 1T‐neuron to control the level of the spiking frequency (*f*). Figure 3d shows the neuronal spiking characteristics of the fabricated 1T‐neuron, which is represented by *V*_out_ versus time. When *I*_in_ was applied to the drain, charges are integrated into the parasitic capacitor because *I*_in_ cannot flow out toward the source. This is the integration process of the LIF neuron, and *V*_out_ measured at the drain was increased by charge integration. At the same time, holes that were generated by iterative impact ionization induced by the increased *V*_D_ accumulate in the body. The integrated charges in the parasitic capacitor are suddenly fired by the STL when *V*_out_ reaches the firing threshold voltage (*V*_T,firing_), which is the same as *V*_latch_. With iterative integration and firing, the spiking characteristics of the biological neuron could be mimicked. It should be noted that when a *V*_G_ of 2 V was applied, integration was not allowed, because *I*_in_ directly flowed out toward the source. In this way, the inhibitory function of the neuron was realized, as shown in Figure S3b,c (Supporting Information). As shown in Figure 3e, *f* linearly increased when *I*_in_ was increased, because the integration speed of the charge in the parasitic capacitor was boosted. In detail, when the resistance of the SMO gas sensor was changed by chemical reactions, *I*_in_ to the 1T‐neuron was accordingly varied. Therefore, *f* of the artificial olfactory neuron module can be modulated by chemoresistance of the SMO sensor. *f* is also varied according to the *C*_par_, as shown in Figure 3f. This was because the increasing speed of *V*_out_ was increased when the *C*_par_ was decreased. Therefore, it is important to select the proper *C*_par_ for the desired frequency level.^23^

### 2.4 Characteristics of an Artificial Olfactory Neuron Module

The *f* of the artificial olfactory neuron module can be modeled as follows:

(1) $$f=\frac{I_{\mathrm{in}}}{C_{\mathrm{par}}V_{\mathrm{pp}}}=\frac{1}{R_{\mathrm{SMO}}C_{\mathrm{par}}ln\left(\frac{V_{\mathrm{DD}}-V_{\mathrm{bottom}}}{V_{\mathrm{DD}}-V_{\mathrm{top}}}\right)}$$

where *V*_pp_ is the peak‐to‐peak output voltage of the 1T‐neuron, *R*_SMO_ is the resistance of the SMO gas sensor, *V*_DD_ is the operating voltage applied to the SMO gas sensor, *V*_bottom_ is the bottom output voltage, and *V*_top_ is the top output voltage of the 1T‐neuron. *V*_top_ is the same as the *V*_T,firing_. Details of the equation are explained in Text S1 (Supporting Information). Since the *R*_SMO_ is changed by the gas species, *f* can be varied because the current that flows into the 1T‐neuron is changed.

**Figure 4:**
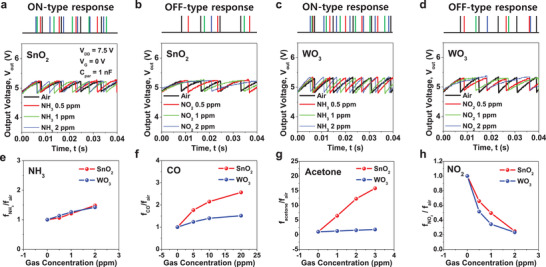
Spiking characteristics of artificial olfactory neuron modules. a,b) Spiking characteristics (*V*_out_–*t*) of the artificial olfactory neuron modules composed of the SnO_2_ gas sensor and the 1T‐neuron. Spiking was activated when the gas concentration of NH_3_ was increased (ON‐type response) and was depressed as the gas concentration of NO_2_ was increased (OFF‐type response). c,d) *V*_out_–*t* of the artificial olfactory neuron modules comprising WO_3_ gas sensor and the 1T‐neuron. Like the SnO_2_‐based neuron module, spiking was activated as the gas concentration of NH_3_ was increased (ON‐type response) and depressed when the gas concentration of NO_2_ was increased (OFF‐type response). e,h) Ratio (*f*_gas_/*f*_air_) for various concentrations of NH_3_, CO, acetone, and NO_2_, where the spiking frequency to gas species is *f*_gas_ and that to air is *f*_air_. Spiking frequency of the artificial olfactory neuron modules was increased in the NH_3_, CO, and acetone environments (ON‐type response), while it was decreased in the NO_2_ environment (OFF‐type response).

Two kinds of artificial olfactory neuron modules were demonstrated, depending on which gas sensor was connected to the 1T‐neuron. One was connected with a SnO_2_ gas sensor and another was connected with the WO_3_ gas sensor. Four target gases (NH_3_, CO, acetone, NO_2_) were used to measure the electrical characteristics of the artificial olfactory neuron modules. The details of the measurement system are provided in Figure S4 (Supporting Information). As shown in **Figure** **4**, *f* of the artificial olfactory neuron modules was changed by different chemoresistance in response to a gas species, being similar to the characteristics of biological olfactory neurons. *V*_DD_ was set to 7.5 V, *V*_G_ was set to 0 V, *P* was set to 12.9 mW, and *C*_par_ was set to 1 nF during the measurement. Referring to Figure 4a,c,,e–g, *f* was increased for both artificial olfactory neuron modules as the concentrations of the NH_3_, CO, and acetone gases were increased. This is because the *R*_SMO_ was decreased as the gas concentration was increased. *f* displayed in Figure 4e–h was averaged for 0.2 s. It should be noted that a single artificial olfactory neuron module alone cannot distinguish every gas, because their corresponding *f* can be similar to each other at certain concentrations. For example, 2 ppm of NH_3_ and 20 ppm of CO could not be distinguished with the single artificial olfactory neuron module composed of the WO_3_ gas sensor and the 1T‐neuron owing to similar responsivities. However, two gases can be identified due to the different responsivities of the two artificial olfactory neuron modules—one with the SnO_2_ gas sensor and another with the WO_3_ gas sensor. Referring to the detection of NO_2_ in Figure 4b,d,,h, *f* decreased as the concentration of NO_2_ was increased for two artificial olfactory neuron modules. This is because the *R*_SMO_ increased as the NO_2_ gas concentration was increased. When the gas sensor was disconnected, *f* from the 1T‐neuron was unchanged, as shown in Figure S5 (Supporting Information). This proves that the changeable *f* according to gas species was attributed to the chemical reaction in the SMO gas sensor. This characteristic indicates that the introduced gas species did not influence the MOSFET‐based 1T neuron.

In summary, the spiking becomes more active as the gas concentration increases for some gases (NH_3_, CO, acetone), and the spiking becomes further inactive as the gas concentration increases for a specific gas (NO_2_). These two cases can be similarly observed in a biological olfactory neuron, where spiking frequency increases or decreases when it is stimulated.^[^ ^51^, ^52^ ^]^ The former with incremental *f* corresponds to an ON‐type response and the latter with decremental *f* corresponds to an OFF‐type response. In addition to the olfactory neuron, this ON‐ and OFF‐type response is a common feature of other sensory neurons, e.g., ganglion cells in the retina. This makes identification easier with a limited number of receptors. In this way, we were able to mimic the biological properties of sensory neurons, and enhance the efficiency of gas identification.

As noted earlier, lateral inhibition in the mitral cell is important for adaptation, signal contrast, and energy efficiency in biological olfactory systems.^[^ ^39^, ^40^, ^41^ ^]^ Neuronal inhibition can be realized by applying the proper voltage to the gate electrode of the 1T‐neuron, as shown in Figure S6 (Supporting Information). When a *V*_G_ of 2 V above the *V*_T_ was applied, high current could flow, regardless of the *V*_D_, as shown in Figure S3a (Supporting Information). This is because a conducting channel was formed by a higher *V*_G_ than the *V*_T_ of the MOSFET.^[^ ^35^, ^36^ ^]^ As a consequence, charge cannot be integrated into the parasitic capacitor and the neuronal spiking is inhibited. In this way, we mimicked the inhibitory function of a biological olfactory neuron to enable lateral inhibition. In a neuromorphic system, it is well known that lateral inhibition can enhance learning and energy efficiency by firing only specific neurons.^[^ ^53^, ^54^ ^]^

To confirm the low power operation of the artificial olfactory neuron module for portable and IoT applications, it is important to reduce the power consumption. Most power was consumed to drive the microheater, and the rest of the power was consumed to enable the olfactory neuron module. The latter power was dominated by the current flowing through the olfactory neuron module composed of the gas sensor and the 1T‐neuron. In order to extract the power consumption, outgoing current flow from the source of the 1T‐neuron was measured, as shown in Figure S7 (Supporting Information). Note that the current only flows at the moment of firing, which is determined by the current of the 1T‐neuron when *V*_latch_ is applied, as shown in Figure 3b. The peak power (*P*_peak_) was calculated to be 165 µW and the average power (*P*_avg_) was calculated to be 350 nW. The detailed procedures to extract *P*_peak_ and *P*_avg_ are explained in Text S2 (Supporting Information). This power consumption for the olfactory neuron module was much lower compared to the power consumption for the conversion circuits to transfer data from the sensor to the processors in an E‐nose chip.^[^ ^55^, ^56^, ^57^ ^]^ Note that *P*_peak_ (not *P*_avg_) is similar to the power consumption of interface circuits and ADCs in the conventional E‐nose chip, although *P*_peak_ includes the power consumed in the sensor part, indicating a large power reduction (Table S2, Supporting Information). However, further development of ultralow power gas sensors will help the reduction of power consumption of the entire gas sensing module, because the major power consumption occurs in the microheater for the SMO gas sensor.^[^ ^58^ ^]^ In addition, the power consumption of the artificial olfactory neuron module can be further reduced by scaling *L*_G_ of the 1T‐neuron.^[^ ^33^, ^34^ ^]^

### 2.5 Semiempirical Simulation for Gas Classification with Software

**Figure 5:**
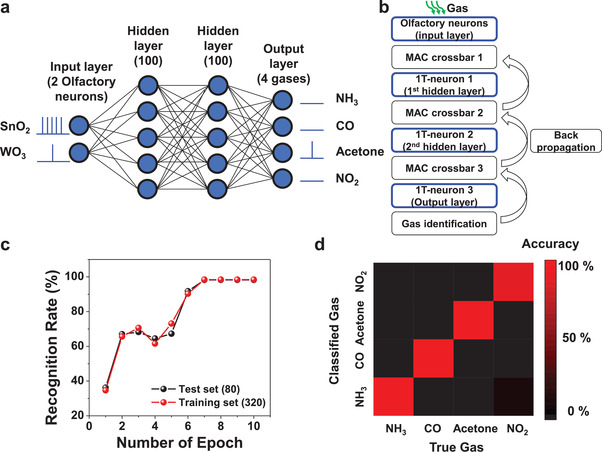
Semi‐empirical software simulation for gas classification. a) Schematic of multi‐layer spiking neural network (SNN) constructed for gas classification. It was composed of two artificial olfactory neurons in the input layer, 100 hidden neurons in two hidden layers each, and four output neurons in the output layer. Two input layers are the neuron module with the SnO_2_ gas sensor and the neuron module with the WO_3_ gas sensor. Measured spiking frequencies of the two artificial olfactory neuron modules were reflected for the semi‐empirical simulations. b) Flow chart of the simulation for the gas classification. The blue boxes represent semi‐empirical simulations for neuronal operations. c) Recognition rate of the test set and training set as a function of the number of epochs. d) Confusion matrix to show the accuracy of test results. The gas classification of the four gases was performed successfully.

Using the measured electrical properties of the artificial olfactory neuron modules, semiempirical simulations with Python software were performed to classify four gases (NH_3_, CO, acetone, NO_2_). A four‐layer SNN was constructed, which was composed of two input neurons, 100 hidden neurons in two hidden layers each, and four output neurons, as shown in **Figure** **5**a. Two input neurons correspond to two artificial olfactory neuron modules for the SNN, one with a SnO_2_ gas sensor and another with a WO_3_ gas sensor. The four output neurons correspond to the abovementioned four gas species. Details of the simulation process are summarized in Figure 5b. The simulation code was modified from the open‐source code produced by Duan.^[^ ^59^ ^]^ Before the simulation, datasets for training and verification were generated by interpolating the measured data, because the measured data were insufficient for effective learning (Figure S8, Supporting Information).^[^ ^60^ ^]^ Each dataset was labeled with an output neuron representing each gas. The measured *f* from the artificial olfactory neuron modules shown in Figure 4 was reflected. The spikes from the input neurons were collected in the first hidden neurons to integrate the weighted signals using a synapse crossbar and to generate spikes when *V*_out_ reached *V*_T,firing_. For the LIF neurons constituting the hidden layers and output neurons, *V*_T,firing_ was set to 5.3 V, and *C*_par_ was set to 1 nF with consideration of the measured electrical characteristics of the 1T‐neuron (Figure 3d). For the synapses constructing the SNN, 128 distinguishable conductance states (= 2^7^ bits) with linearity and symmetry were set for potentiation and depression. The generated spikes were propagated to the next synapse crossbars and neuron layers, and these processes were repeated in each layer of the SNN. Last, the spiking of the output neurons was detected to identify the gas species. By comparing the classified results with the expected results, the SNN was trained by backpropagation. Instead of the step function of the spiking neuron, a sigmoid activation function was utilized to obtain the gradient during the backward propagation of errors, and then the synaptic weights were modified accordingly.

Figure 5c shows the classification accuracy as a function of the training epochs. Training was performed with 320 training datasets and validation was performed with 80 test datasets. The training and test datasets were randomly selected from the interpolated data in Figure S8 (Supporting Information). As a result, gas classification was successful using the artificial olfactory neuron modules with an accuracy of 98.25% after seven epochs. Figure 5d displays a confusion matrix of the classified results from the 80 test datasets. It lists the expected results according to the classified results. A colored scale bar represents the accuracy of the classification. As a result, it is concluded that four different gas species were well classified after the training.

### 2.6 Hardware Implementation for Wine Classification

To show a further application of the artificial olfactory neuron modules, we prepared a fully hardware‐based E‐nose for wine classification. Wine is one of the most popular beverages in the world. With such a large consumer market, wine classification and evaluation have become more important to preserve quality and economic value, and to prevent mislabeling and illegal labeling.^[^ ^61^, ^62^ ^]^ Aroma is an important indicator used to classify wines and is strongly linked to wine characteristics because wines contain several volatile organic compounds, which are typically reducing gases. However, distinguishing wines is challenging due to the complexity and diversity of the compounds. The E‐nose has advantages in complex pattern recognition, which has been considered a useful solution for wine classification.^[^ ^63^, ^64^ ^]^ In addition, compact hardware and low energy consumption would allow the portability of a wine testing device for such applications.

**Figure 6:**
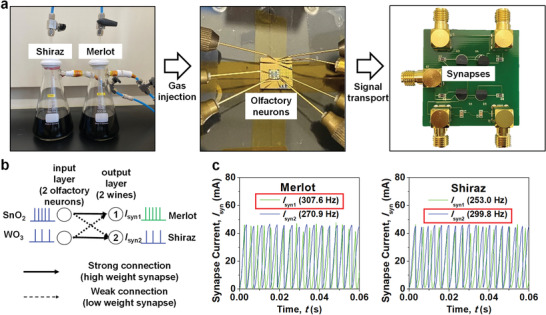
Hardware implementation for wine classification. a) Experimental setup used for wine classification. Wine gas was injected into a probe box where the artificial olfactory neuron modules were located. Electrical signals from the artificial olfactory neuron modules were transported to synapses mounted on the printed circuit board (PCB). b) Neural network based on a single‐layer spiking neural network (SNN) for the classification of two wines. The input layers corresponded to the two artificial olfactory neuron modules and the output layers corresponded to the two wines. c) Synapse current collected at the output layer ① (*I*_syn1_) and at the output layer ② (*I*_syn2_). For “Merlot” wine, the spiking frequency of *I*_syn1_ was larger than *I*_syn2_. Otherwise, the result was reversed; i.e., spiking frequency of *I*_syn2_ > *I*_syn1_ for “Shiraz” wine.

We constructed E‐nose hardware based on a single‐layer SNN, to classify two wines, “Shiraz” and “Merlot,” which are made with different red grape varieties. Figure S9 (Supporting Information) shows the decrease of resistance of the gas sensors when the sensors reacted with the wine gases since they are composed of reducing gases. In Section 2.5, it was confirmed that the combination of artificial olfactory neurons and a spiking neural network (SNN) allows for the identification of different types of reducing gases. Therefore, it was conjectured that the same method can be applied to the classification of different wines. **Figure** **6**a shows the experimental setup including the wines, input neurons (artificial olfactory neuron modules), and synapses. Each wine was placed in a sealed flask to balance the air inside the flask, and the liquid state of the wine was changed to a gas state by injecting dry nitrogen gas into the flask through mass flow controllers. This is to suppress a possible humidity effect. The mass flow rates of the nitrogen gas and the wine gas were 499 and 1 sccm, respectively. As a consequence, the relative humidity (RH) of the injected wine gas could not exceed 0.2%, which is small enough to exclude the humidity effect. The wine gas was then introduced into the probe box, where the artificial olfactory neuron modules composed of the SMO gas sensor and the 1T‐neuron were located. The output voltage of the neurons was transported to the synapses to reflect the synaptic weight. The synapses were constructed with a hybrid of a stand‐alone transistor and a single‐typed resistor (1T1R), one of the most popular synaptic structures.^[^ ^65^, ^66^ ^]^ For the synapses, commercial stand‐alone MOSFET chips (VN0550N3‐G, Microchip) and resistors were mounted on a printed circuit board (PCB). Capacitors were also implemented on the PCB to control the spiking frequency.

Figure 6b shows the neural network designed for wine classification. It was composed of an input layer with two artificial olfactory neuron modules (1T‐neuron with SnO_2_ gas sensor and 1T‐neuron with WO_3_ gas sensor) and an output layer corresponding to the two wines. The neuron module with a SnO_2_ gas senor was strongly connected to the output layer ① and weakly to the output layer ②. Meanwhile, the neuron module with the WO_3_ gas sensor was strongly linked to the output layer ② and weakly to the output layer ①. The strong and weak connections were represented with the binary weights of the synapses, by changing the resistance of the resistors in the 1T1R structure. A resistance of 10 Ω was used for the high weight synapse (strong connection) and 10 kΩ was used for the low weight synapse (weak connection). As a result, simple E‐nose hardware composed of two SMO gas sensors, two 1T‐neurons, two capacitors, four MOSFETs, and four resistors was constructed, as depicted in the circuit diagram of Figure S10 (Supporting Information). Finally, the synapse current collected at the output layer ① (*I*_syn1_) and at the output layer ② (*I*_syn2_) was measured to classify the wine. As shown in Figure 6c, *I*_syn1_ and *I*_syn2_ showed a spike‐shaped output because the gate of the MOSFET in the 1T1R synapse received the spike‐shaped output voltage from the artificial olfactory neuron modules. It was possible to classify the wine depending on which spiking frequency was larger, *I*_syn1_ or *I*_syn2_. Specifically, the wine could be classified as “Merlot” when *I*_syn1_ had a larger spiking frequency, and as “Shiraz” when *I*_syn2_ showed larger spiking frequency. For example, when the wine was “Merlot,” the spiking frequency of *I*_syn1_ was 307.6 Hz and that of *I*_syn2_ was 270.9 Hz. Otherwise, when the wine was “Shiraz”, the spiking frequency of *I*_syn1_ was 253.0 Hz and that of *I*_syn2_ was 299.8 Hz. It should be noted that when the firing of one olfactory neuron module is observed, the firing of the other olfactory neuron module can be inhibited similar to the biological lateral inhibition, to enhance the signal contrast and the energy efficiency, as shown in Figure S11 (Supporting Information). This demonstration confirmed that two wines could be classified using the E‐nose hardware, which included artificial olfactory neuron modules. For classification of more various kinds of wine, an increased number of multiple‐layer SNNs can be used. Note that the proposed E‐nose hardware can only perform the inference function. In order to perform the training function by the weight update, a resistor used for an 1T‐1R synapse can be replaced by a resistance‐tunable synaptic device such as memristor.

## 3 Conclusion

In summary, we have proposed an artificial olfactory neuron module for application to a neuromorphic E‐nose. A biological olfactory neuron was emulated using a SMO gas sensor and an 1T‐neuron, wherein the spiking frequency was varied depending on the gas species. In addition, important functions of the biological olfactory neuron required for efficient odor classifications were demonstrated, such as ON‐ and OFF‐type responses and the inhibitory function. The proposed device can perform gas detection and spike encoding simultaneously, actions that are required for the input neuron of the internal SNN in the sensor module. This can greatly reduce hardware cost and power consumption by eliminating the heavy and energy‐consuming signal transducer components, such as interface circuits and the ADC. In addition, a powerful computer with hardware components such as CPU and memory needed for von Neumann computing is no longer required, thanks to the bioinspired neuromorphic architecture, which can also greatly reduce the hardware cost and power consumption. Using the fabricated artificial olfactory neuron modules, four different gases were successfully classified (98.25%) with the aid of experiment‐based software simulations. Beyond the gas identification with semi‐empirical simulations, two different wines were classified with pure neuromorphic hardware, which can serve as an electronic sommelier. Because the proposed neuromorphic E‐nose is highly scalable and energy efficient, it will open a new avenue for mobile gas sensors and IoT applications to monitor air quality, toxic gases, food quality, and so on.

## 4 Experimental Section

### Fabrication of the SMO Gas Sensor

SnO_2_ and WO_3_ were used as the sensing materials and were deposited by glancing angle deposition via RF sputtering to form the nanocolumnar thin films. A MEMS‐based suspended microheater platform was used for the SMO gas sensor. A detailed description of the fabrication process of the SMO gas sensor is provided in Figure S1 (Supporting Information).

### Fabrication of the 1T Neuron

A 1T‐neuron with a channel width (*W*) of 200 nm, a gate length (*L*_G_) of 1900 nm, and an equivalent oxide thickness (EOT) of 13 nm was fabricated on an SOI wafer using a standard MOSFET fabrication process. See Figure S2 (Supporting Information) for details of the fabrication process.

### Electrical Characterization

The electrical characteristics of the artificial olfactory neuron module and a 1T‐neuron were measured using a 4156C semiconductor parameter analyzer (Keysight). The electrical characteristics of the SMO sensor were measured using a SMU 2400 source meter (Keithley) and an E3652A DC power supply.

### SEM Analysis

SEM images were taken using a Magellan 400 field emission SEM (FEI company).

### Gas Test

Gas tests were performed under atmospheric pressure in a gas chamber using mass flow controllers (ATOVAC, Korea) that can control the concentrations of each gas. The total gas flow rate injected into the gas chamber was set to 500 sccm, and the gas tests were performed at room temperature since the sensing materials were heated by using microheaters. See Figure S4 (Supporting Information) for the detailed measurement system.

### Software‐Based Simulation

Software simulations for the gas classification were performed using Python. Measured spiking frequency to each gas species via the artificial olfactory neuron modules was reflected in the simulations.

## Conflict of Interest

The authors declare no conflict of interest.

## Supporting information

Supporting Information

## Data Availability

The data that support the findings of this study are available from the corresponding author upon reasonable request.
