# Supplementary material for: Artificial Olfactory Neuron for an In‐Sensor Neuromorphic Nose
Source: Adv Sci (Weinh). 2022 Apr 15;9(18):2106017. doi: 10.1002/advs.202106017 (PMC9218653; doi:10.1002/advs.202106017)
Supplement: Supplementary file 1 — Supporting Information [file ADVS-9-2106017-s001.pdf]

ADVANCED SCIENCE
Open Access

Supporting Information

for *Adv. Sci.*, DOI 10.1002/advs.202106017

Artificial Olfactory Neuron for an In-Sensor Neuromorphic Nose

Joon-Kyu Han, Mingu Kang, Jaeseok Jeong, Incheol Cho, Ji-Man Yu, Kuk-Jin Yoon, Inkyu Park and Yang-Kyu Choi**

Supporting Information

Artificial olfactory neuron for an in-sensor neuromorphic nose

Joon-Kyu Han†, Mingu Kang†, Jaeseok Jeong, Incheol Cho, Ji-Man Yu, Kuk-Jin Yoon, Inkyu Park and Yang-Kyu Choi**

J.-K. Han, J.-M. Yu, Y.-K. Choi
School of Electrical Engineering, Korea Advanced Institute of Science and Technology (KAIST), 291 Daehak-ro, Yuseong-gu, Daejeon 34141, Republic of Korea

M. Kang, J. Jeong, I. Cho, K.-J. Yoon, I. Park
Department of Mechanical Engineering, Korea Advanced Institute of Science and Technology (KAIST), 291 Daehak-ro, Yuseong-gu, Daejeon 34141, Republic of Korea

†These authors equally contributed to this work.

E-mail: ykchoi@ee.kaist.ac.kr and inkyu@kaist.ac.kr

Supplementary text

1. Spiking frequency (*f*) equation of the artificial olfactory neuron module

The relation between input current ($I_{\rm in}$) and the output voltage ($V_{\rm out}$) of the 1T-neuron can be expressed with the following equation:

$$I_{in} = C_{par}\frac{dV_{out}}{dt}\ (1),$$

where $C_{\rm par}$ is the parasitic capacitance connected in parallel to the 1T-neuron. By using the resistance of the SMO gas sensor ($R_{\rm SMO}$) to represent $I_{\rm in}$, the spiking frequency (f) of the artificial olfactory neuron module can be expressed using the following equations:

$$\int_0^{\frac{1}{f}} dt = \int_{V_{\rm bottom}}^{V_{\rm top}} \frac{C_{\rm par}}{I_{\rm in}} dV_{\rm out} = \int_{V_{\rm bottom}}^{V_{\rm top}} \frac{R_{\rm SMO}C_{\rm par}}{(V_{\rm DD}-V_{\rm out})} dV_{\rm out} = R_{\rm SMO}C_{\rm par} ln(\frac{V_{\rm DD}-V_{\rm bottom}}{V_{\rm DD}-V_{\rm top}})\ (2)$$

$$f = \frac{1}{R_{\rm SMO}C_{\rm par} ln(\frac{V_{\rm DD}-V_{\rm bottom}}{V_{\rm DD}-V_{\rm top}})}\ (3),$$

where $V_{\rm top}$ is the top voltage of $V_{\rm out}$, $V_{\rm bottom}$ is the bottom voltage of $V_{\rm out}$, and $V_{\rm DD}$ is the operating voltage applied to the SMO gas sensor. As a result, it was confirmed that f is inversely proportional to $R_{\rm SMO}$.

2. Power consumption of the artificial olfactory neuron module

Two kinds of power consumption were extracted for the artificial olfactory neuron module. The first is the peak power consumption ($P_{\rm peak}$), which is the power consumed at the moment of firing. Thus, $P_{\rm peak}$ is simply estimated by the product of $I_{\rm peak}\cdot V_{\rm DD}$, where $I_{\rm peak}$ is the peak current. From the measured $I_{\rm peak}$ shown in **Figure S8**, $P_{\rm peak}$ of 165 μW was extracted for the artificial olfactory neuron module composed of the SnO_2 gas sensor and the 1T-neuron at NH_3 of 0.5 ppm. The second is the average power consumption ($P_{\rm avg}$), which is the average power consumed in one spiking cycle. It can be extracted by integrating the current using the following equation: $P_{\rm avg} = f \cdot V_{DD} \cdot \int_0^{\frac{1}{f}} I\, dt$. As a result, $P_{\rm avg}$ of 350 nW was extracted.

Supplementary figures

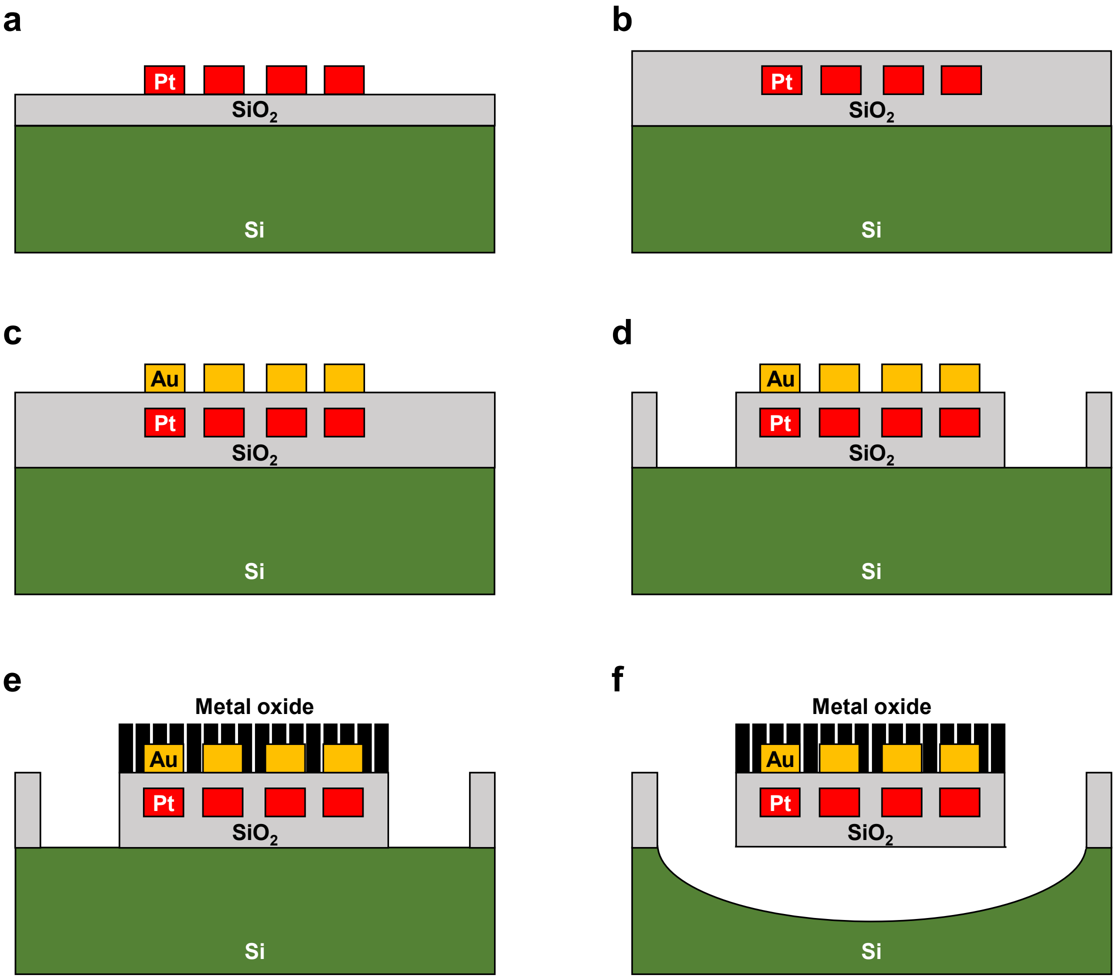

Figure S1. Fabrication procedure for a SMO gas sensor. a) A SiO_2 layer of 1 μm thickness was deposited on a Si wafer using the plasma-enhanced chemical vapor deposition (PECVD) process, and Ti/Pt (10 nm/200 nm thickness) for microheaters were deposited and patterned on the SiO_2 by E-beam evaporation and lift-off. b) A SiO_2 layer of 800 nm thickness was deposited by PECVD for electrical insulation between the sensing electrodes and the microheaters, and the insulation layer on probing pads of the heater was selectively etched by buffered oxide etchant (BOE). c) Cr/Au (10 nm/200 nm thickness) for interdigitated sensing electrodes were deposited and patterned on the insulation layer by E-beam evaporation and lift-off. d) The SiO_2 was etched by reactive ion etching (RIE) to form the etched Si window required for the bridge-typed microheater. e) SnO_2 or WO_3 thin films were separately deposited on the sensing electrodes by glancing angle deposition *via* RF sputtering, and Au nanoparticles with a deposition thickness of 1 nm were additionally deposited on the metal

oxide thin films by E-beam evaporation. The Au decorated metal oxide films were patterned by lift-off, and thermal annealing was performed in N_2 at 400°C for 2 hours. f) The Si substrate located under the microheater was etched away by XeF_2 vapor etching.

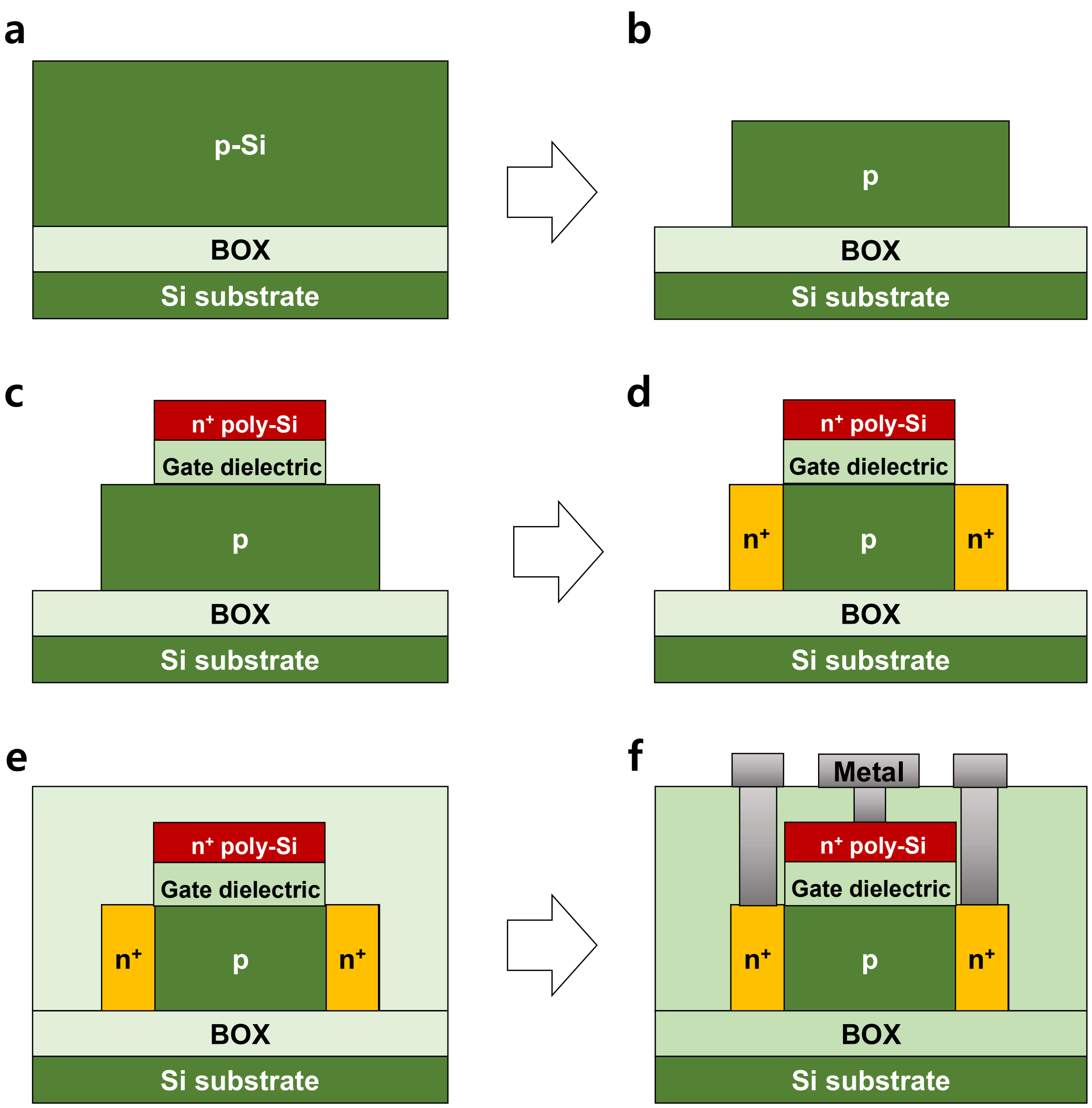

Figure S2. Fabrication procedure for a 1T-neuron. a) A p-type (100) SOI wafer with a buried-oxide (BOX) thickness of 140 nm was used as a starting wafer. b) The top Si was thinned down to 50 nm and the active area was patterned to define the channel width (*W*), using photo-lithography and plasma etching. Body doping with boron implantation (1.4×10^{13} cm^{-3}, 10 keV) and rapid thermal annealing (1000 °C, 5 sec) were then carried out. c) A gate dielectric with an equivalent oxide thickness (EOT) of 13 nm and a gate electrode of *in-situ* doped n^+ poly-Si with an 100 nm thickness were stacked. The gate area was then patterned by photo-lithography and plasma etching to define the gate length (L_G). d) Source/drain doping

with arsenic implantation (1×10^{16} cm^{-2}, 55 keV) and rapid thermal annealing (1000 °C, 5 sec) were performed. e) An inter-layer dielectric (ILD) of 300 nm was deposited with tetraethyl orthosilicate (TEOS) by low-pressure chemical vapor deposition (LPCVD). f) Metallization was performed with Ti/TiN/Al (10 nm/20 nm/500 nm) after the contact etch.

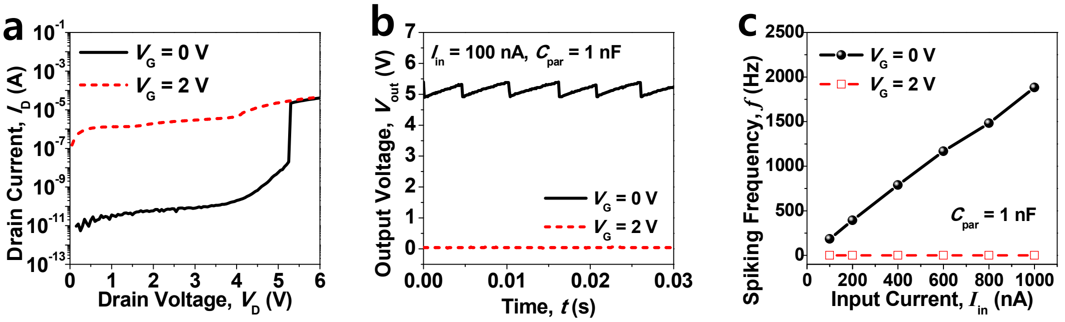

Figure S3. Inhibitory function of 1T-neuron. a) Output characteristics (I_D-V_D) of the 1T-neuron depending on applied gate voltage (V_G). When a V_G of 2 V was applied, single transistor latch (STL) was disabled due to large current flowing regardless of the drain voltage (V_D). b) Spiking characteristics (V_{out}-t) depending on the applied V_G. Neuronal spiking was inhibited for a V_G of 2 V. C) Spiking frequency (f) versus input current (I_{in}) depending on the applied V_G. When a V_G of 2 V was applied, f was extracted as zero regardless of I_{in} owing to inhibited firing.

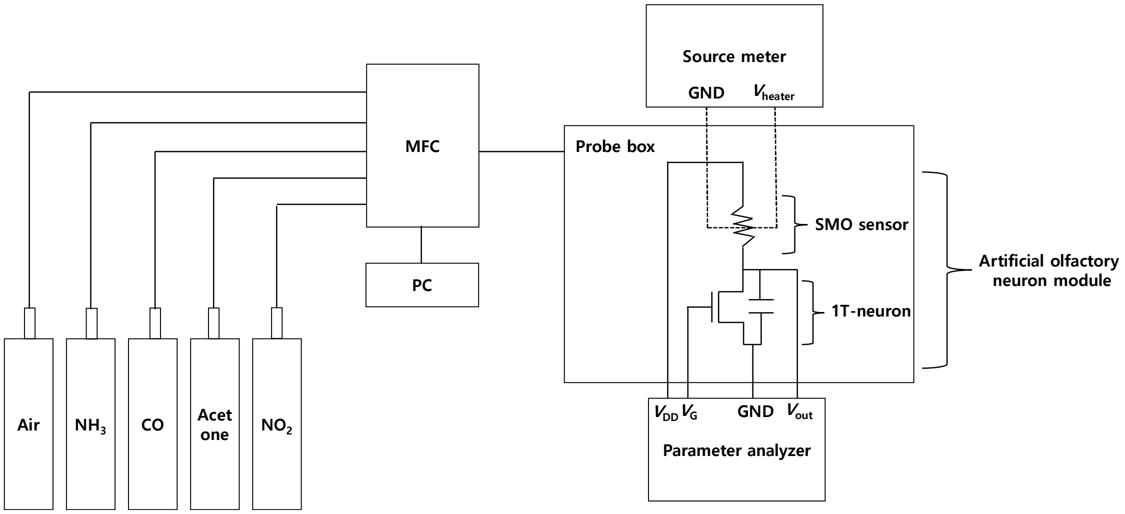

Figure S4. Measurement setup for artificial olfactory neuron module. The measurement apparatus for the artificial olfactory neuron module was composed of gas chambers (air, NH_3, CO, acetone, NO_2), a mass flow controller (MFC) to control the gas concentrations, a source meter, a parameter analyzer, and a probe box to enclose the artificial olfactory neuron module.

The source meter supplies power to the microheater underneath the SMO gas sensor. The parameter analyzer applies voltage to the SMO gas sensor (variable resistor) and the 1T-neuron, and measures the output voltage (V_{out}).

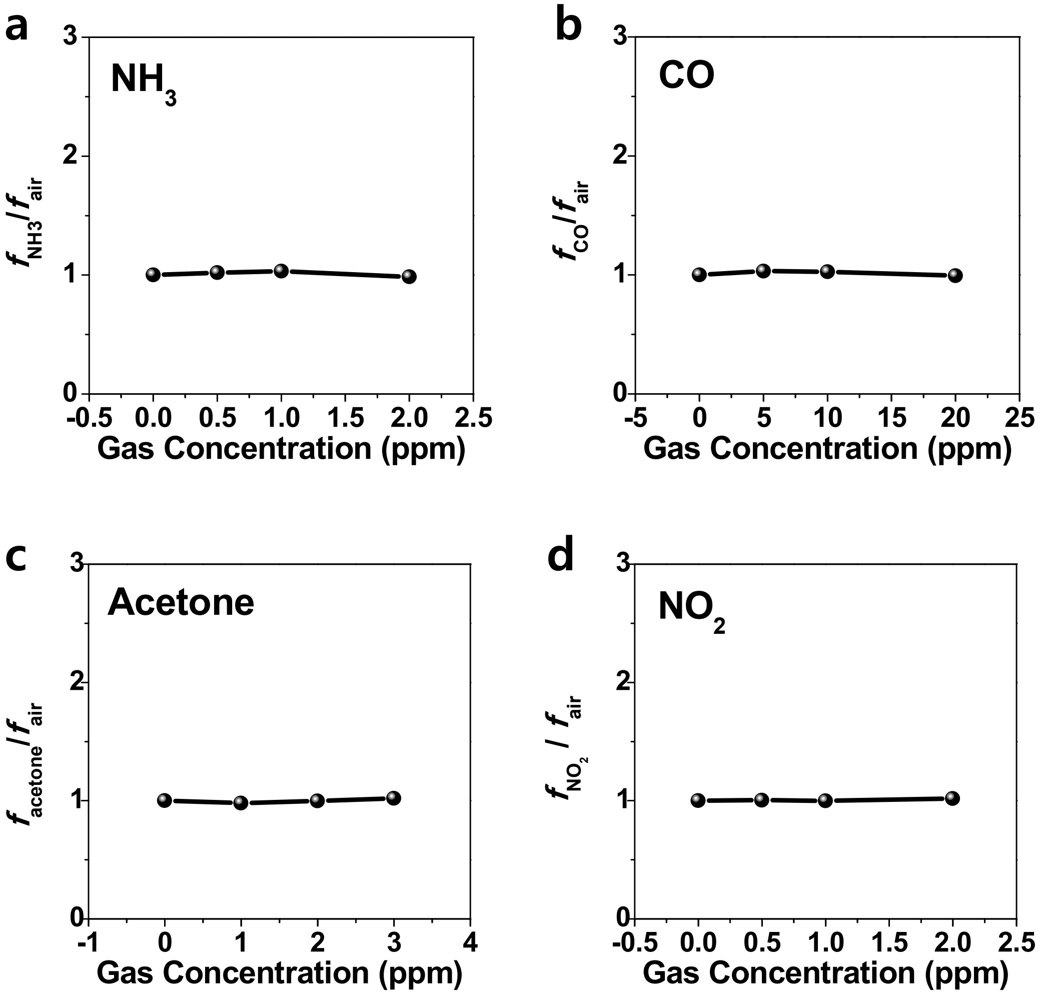

Figure S5. Gas independent spiking characteristics of disconnected 1T-neuron. Ratio of spiking frequency in a gas environment (f_{gas}) and spiking frequency in air environment (f_{air}) depending on the gas concentration of a) NH_3 b) CO c) acetone d) NO_2. For all gases, f_{gas}/f_{air} was not varied. Thus, the introduced gases into the probe box did not affect the MOSFET characteristics in the 1T-neuron.

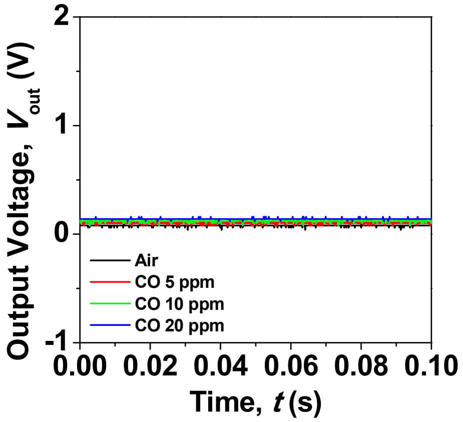

Figure S6. Inhibition of the artificial olfactory neuron module. The spiking characteristics (V_{out}-t) of the artificial olfactory neuron module when a V_G of 2 V was applied for inhibition. The measurement was performed with the artificial olfactory neuron module composed of the SnO_2 gas sensor and the 1T-neuron. Regardless of the CO concentrations, neuronal spiking was inhibited. In a biological olfactory system, lateral inhibition of the mitral cell controlled by interneurons in the olfactory bulb is important for adaptation and signal contrast.

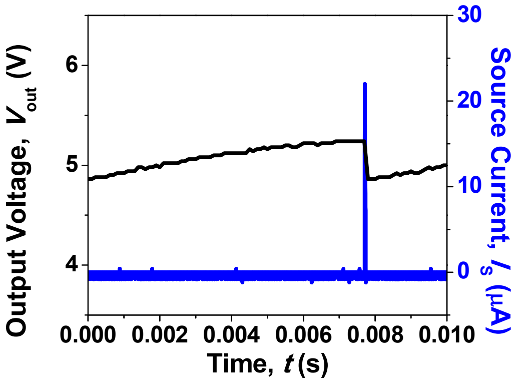

Figure S7. Power consumption of the artificial olfactory neuron module. In order to determine the power consumption of the artificial olfactory neuron module, outgoing source current (I_S) from the source of the 1T-neuron was measured. The measurement was performed with the artificial olfactory neuron module composed of the SnO_2 gas sensor and the 1T-neuron, in a gas environment of NH_3 (0.5 ppm). Extracted P_{peak} was 165 μW when firing occurred. There was no power consumption during the integration process.

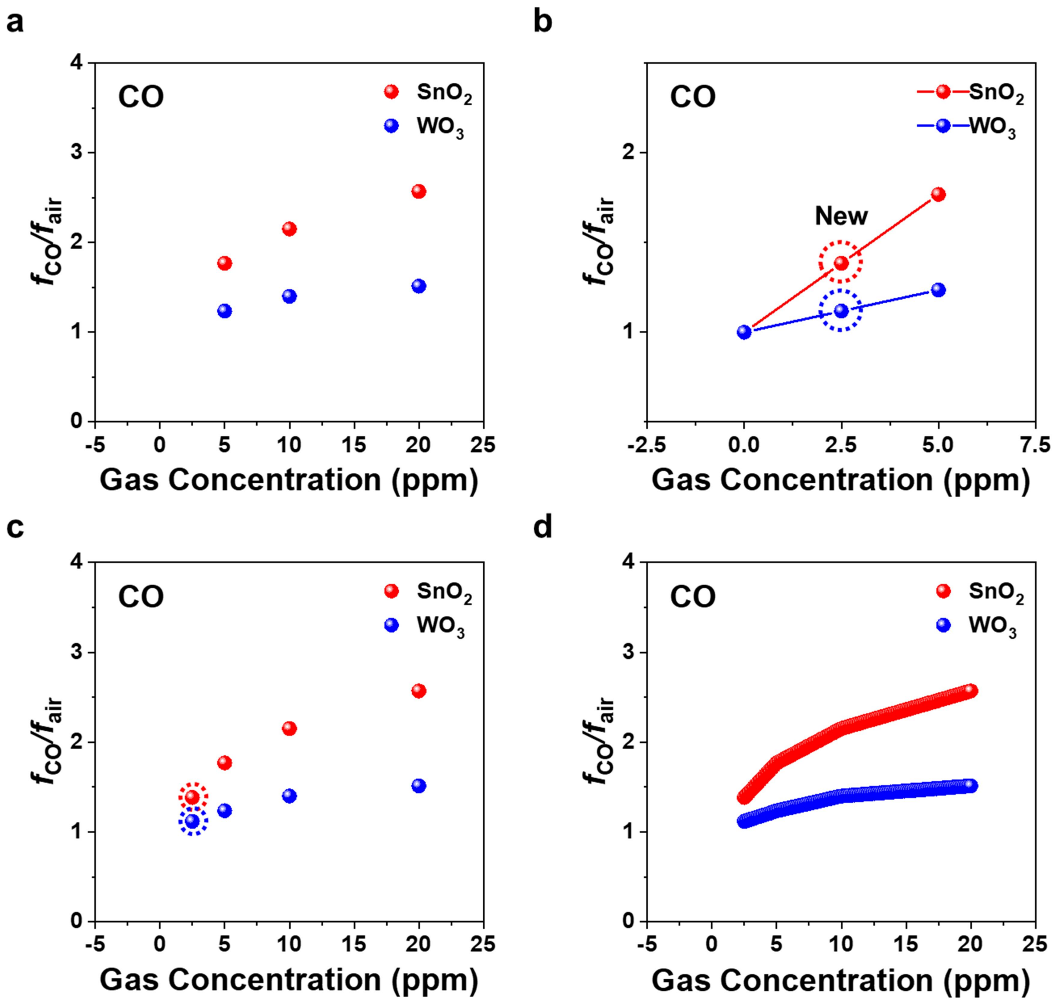

Figure S8. Datasets for software simulations. a) Extracted $f_{gas(=CO)}/f_{air}$ for CO gas. b) Interpolated f_{CO}/f_{air} for a range of CO concentration from 0 ppm to 5 ppm. c) 4 datasets of the spiking characteristics from the olfactory neuron modules containing one generated value. d) Extended 400 datasets through linear interpolation based on three measured datasets and one interpolated dataset. The same process was carried out for the NH_3, acetone, and NO_2 gases. 320 datasets were used for training and 80 datasets were used for validation.

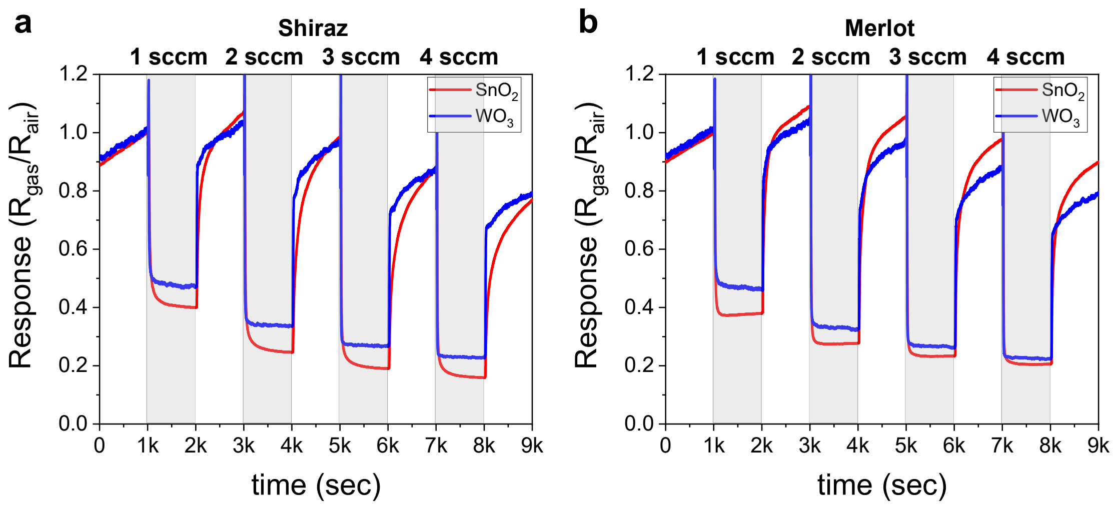

Figure S9. Dynamic responses of SnO_2 and WO_3 gas sensors to the wine gases. Responses to a) Shiraz and b) Merlot. Shaded and unshaded areas represent time intervals exposed to the wine gas and to the air, respectively. The total gas flow rate injected into the gas chamber was set to 500 sccm, and the wine gases were injected with a flow rate from 1 sccm to 4 sccm. Both wine gases have reducing gas properties.

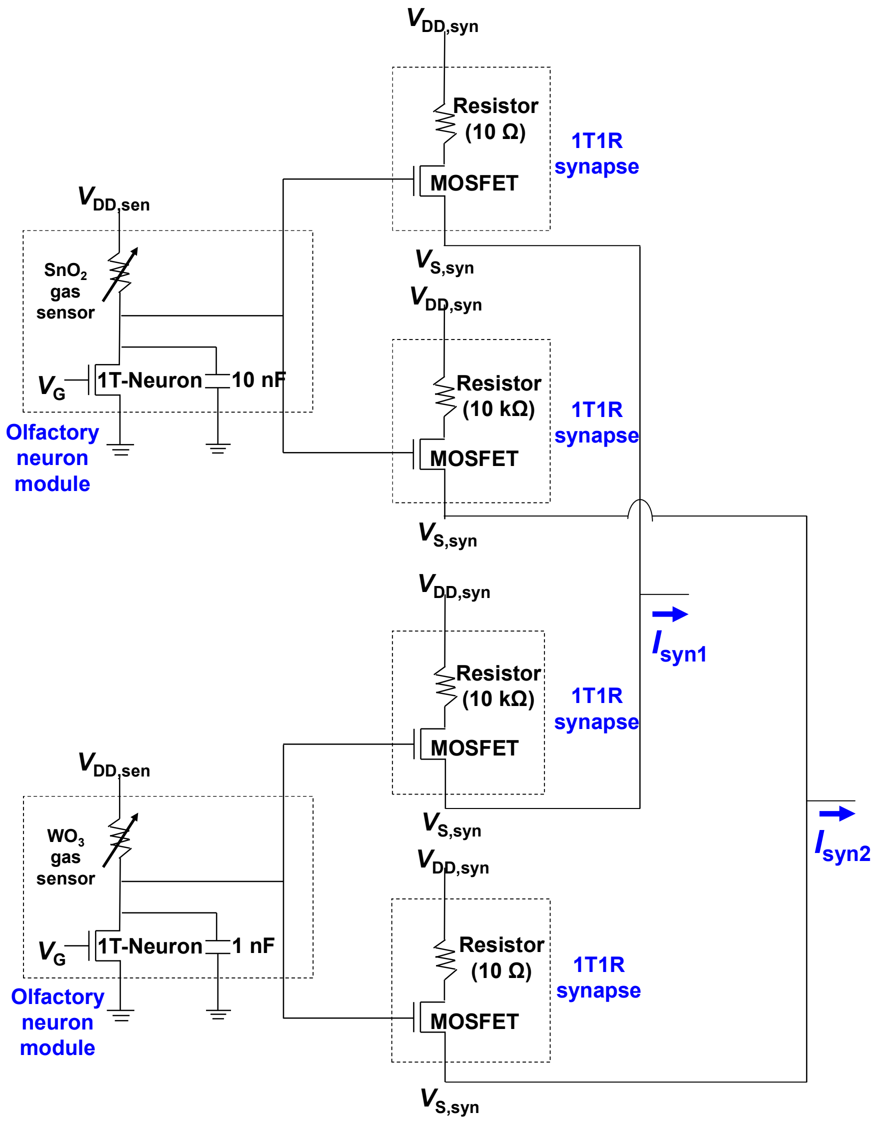

Figure S10. Circuit diagram of the E-nose hardware used for wine classification. The circuit includes two artificial olfactory neuron modules that act as input neurons. One artificial olfactory neuron module was composed of the SnO_2 gas sensor and one 1T-neuron, while the other artificial olfactory neuron module was composed of the WO_3 gas sensor and the other 1T-neuron. Capacitors were connected to control the spiking frequency. The capacitor connected to the SnO_2 gas sensor was larger so as to decrease the spiking frequency, because it was found that the responsivity of the SnO_2 gas sensor to the wines was larger than the responsivity of the WO_3 gas sensor. The voltages applied to the sensor ($V_{DD,sen}$) and to the gate of the 1T-neuron (V_G) were set at 7.5 V and 0 V, respectively. The synapses had a 1T1R structure, which comprised a commercial stand-alone MOSFET and a single-typed resistor. The high weight synapse had resistance of 10 Ω and the low weight synapse had resistance of 10 kΩ. The voltages applied to the resistor in the 1T1R synapse ($V_{DD,syn}$) and to the source of

the MOSFET in the 1T1R synapse ($V_{S,syn}$) were 5 V and 3 V, respectively. The output currents from the synapses (I_{syn}) were measured to compare the spiking frequency of I_{syn1} and I_{syn2}.

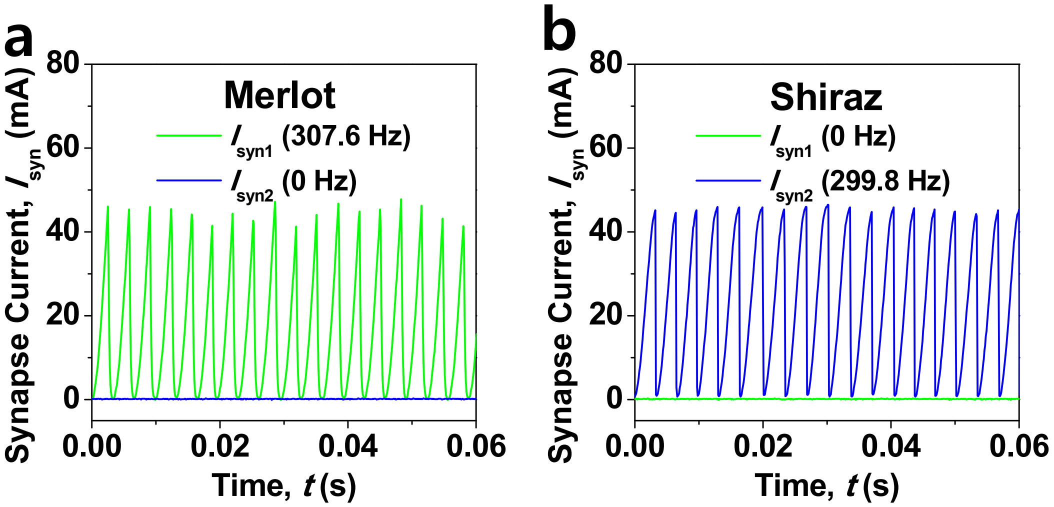

Figure S11. Lateral inhibition for wine classification. The synapse current collected at output layer ① (I_{syn1}) and at output layer ② (I_{syn2}) when the lateral inhibition was applied. a) When the wine was 'Merlot', the artificial olfactory neuron module composed of WO_3 and the 1T-neuron was inhibited by applying a V_G of 2 V. b) When the wine was 'Shiraz', the artificial olfactory neuron module comprising SnO_2 and the 1T-neuron was inhibited. Such lateral inhibition can enhance signal contrast and the energy efficiency by firing of only a specific neuron.

Supplementary table

1. Comparison with previously reported artificial sensory neurons

Ref.	Architecture	Sensing
[S1]	Thermogenerator + diffusive memristor	Temperature (temperature sensor)
[S2]	Piezoelectric generator + Mott memeristor	Pressure (tactile sensor)
[S3]	Single transistor	Visible light (visual sensor)
[S4]	UV sensor + Mott memristor	UV light (visual sensor)
This work	**SMO gas sensor + single transistor**	**Gas (olfactory sensor)**

2. Comparison of power consumption with the conversion circuit in conventional E-nose chip

Ref.	Power
[S5]	42.3~329.1 μW @ interface circuits<br>2.86 μW @ ADC
[S6]	401.899 μW @ interface circuits<br>19.65 μW @ ADC
[S7]	53.4~111.4 μW @ interface circuits<br>5.14 μW @ ADC
This work	**350 nW (average)**<br>**165 μW (peak)**

References

[S1] J. H. Yoon, Z. Wang, K. M. Kim, H. Wu, V. Ravichandran, Q. Xia, C. S. Hwang, J. J. Yang, *Nat. Commun.* **9**, 1-9 (2018).

[S2] X. Zhang, Y. Zhuo, Q. Luo, Z. Wu, R. Midya, Z. Wang, W. Song, R.Wang, N. K.Upadhyay, Y. Fang, F. Kiani, M. Rao, Y. Yang, Q. Xia, Q. Liu, M. Liu, J. J. Yang, *Nat. Commun.* **2020**, *11*, 51.

[S3] J.-K. Han, D.-M. Geum, M.-W. Lee, J.-M. Yu, S. K. Kim, S. Kim, Y.-K. Choi, *Nano Letters* **2020**, *20*, 8781.

[S4] Q. Wu, B. Dang, C. Lu, G. Xu, G. Yang, J. Wang, X. Chuai, N. Lu, D. Geng, H. Wang, L. Li, *Nano Letters* **2020**, *20*, 8015.

[S5] T K.-T. Tang, S.-W. Chiu, M.-F. Chang, C.-C. Hsieh, J.-M. Shyu, *IEEE J. Solid-State Circuits* **2011**, *39*, 661.

[S6] S.-W. Chiu, J.-H. Wang, G.-T. Lin, C.-L. Chang, H. Chen, K.-T. Tang, *Proc. IEEE 55th Int. Midwest Symp. Circuits and Systems*, **2012**, 166.

[S7] S.-W. Chiu, H.-C. Wu, T.-I. Chou, H. Chen, K.-T. Tang, *Analytical and Bioanalytical Chemistry* **2014**, *406*, 3985.
